# Neurobeachin Regulates Glutamate- and GABA-Receptor Targeting to Synapses via Distinct Pathways

**DOI:** 10.1007/s12035-015-9164-8

**Published:** 2015-05-02

**Authors:** F. Farzana, R. Zalm, N. Chen, K. W. Li, Seth G. N. Grant, A. B. Smit, R. F. Toonen, M. Verhage

**Affiliations:** Department of Functional Genomics, Centre for Neurogenomics and Cognitive Research (CNCR), Neuroscience Campus Amsterdam, VU Medical Centre, VU University Amsterdam, 1081HV Amsterdam, The Netherlands; Department of Molecular and Cellular Neurobiology, Centre for Neurogenomics and Cognitive Research, Neuroscience Campus Amsterdam, VU University Amsterdam, 1081HV Amsterdam, The Netherlands; Centre for Clinical Brain Sciences, Edinburgh University, Edinburgh, EH16 4SB United Kingdom; Department of Clinical Genetics, Centre for Neurogenomics and Cognitive Research, Neuroscience Campus Amsterdam, VU Medical Centre, VU University Amsterdam, Amsterdam, The Netherlands

**Keywords:** Synapse, Neurobeachin, AKAP, SAP102, Glutamate receptors, GABA receptors

## Abstract

Neurotransmission and synaptic strength depend on expression of post-synaptic receptors on the cell surface. Post-translational modification of receptors, trafficking to the synapse through the secretory pathway, and subsequent insertion into the synapse involves interaction of the receptor with A-kinase anchor proteins (AKAPs) and scaffolding proteins. Neurobeachin (Nbea), a brain specific AKAP, is required for synaptic surface expression of both glutamate and GABA receptors. Here, we investigated the role of Nbea-dependent targeting of postsynaptic receptors by studying Nbea interaction with synapse-associated protein 102 (SAP102/Dlg3) and protein kinase A subunit II (PKA II). A Nbea mutant lacking the PKA binding domain showed a similar distribution as wild-type Nbea in *Nbea null* neurons and partially restored GABA receptor surface expression. To understand the relevance of Nbea interaction with SAP102, we analysed *SAP102* null mutant mice. Nbea levels were reduced by ~80 % in *SAP102* null mice, but glutamatergic receptor expression was normal. A single-point mutation in the pleckstrin homology domain of Nbea (E2218R) resulted in loss of binding with SAP102. When expressed in *Nbea* null neurons, this mutant fully restored GABA receptor surface expression, but not glutamate receptor expression. Our results suggest that the PKA-binding domain is not essential for Nbea’s role in receptor targeting and that Nbea targets glutamate and GABA receptors to the synapse via distinct molecular pathways by interacting with specific effector proteins.

**Electronic supplementary material:**

The online version of this article (doi:10.1007/s12035-015-9164-8) contains supplementary material, which is available to authorized users.

## Introduction

The number of functional neurotransmitter receptors in the postsynaptic membrane is the dominant factor in the regulation of synaptic strength. Receptor abundance and mobility in the postsynaptic membrane are regulated by different mechanisms, such as constitutive and activity dependent insertion, local mobility such as recycling via endo/exocytosis and lateral diffusion from extra-synaptic sites [1–3]. Membrane-associated guanylate kinases (MAGUKs) in the discs large family (Dlg) of scaffolding proteins are central in the regulation of receptor abundance and mobility in excitatory neurons [4]. Mutations of Dlg proteins in mice and humans result in cognitive disorders and impairments in synaptic plasticity [5–7]. These scaffold proteins link NMDA and AMPA receptors with signalling and structural proteins in postsynaptic multiprotein complexes. Evidence suggests that MAGUKs assemble with the receptor subunits during biosynthesis in the Golgi and when this interaction is disturbed, NMDAs do not reach the synapse [8–10]. However, the principles of receptor assembly and targeting to the synapse are largely unknown.

Another class of scaffold proteins playing an important role in excitatory synapses are A-kinase anchoring proteins (AKAP), which bind the regulatory subunit of protein kinase A (PKA). In mice lacking the AKAP protein neurobeachin (Nbea), synaptic transmission is severely impaired [11], and they die shortly after birth [12]. Loss of Nbea results in reduced spine number in cultured neurons [13] and modifications in the composition of synaptic proteins [14]. Nbea binds to the MAGUK protein synapse-associated protein 102 (SAP102/Dlg3) [15] and the type II regulatory subunit of protein kinase A (PKA II) [16]. Thus, Nbea may be involved in recruiting and targeting PKA to specific substrate proteins. Synaptic surface expression of both inhibitory (GABAergic) and excitatory receptors (NMDA, AMPA and kainate) are decreased by Nbea loss, suggesting that Nbea controls a generic principle of receptor targeting, despite the fact that the mechanisms of receptor targeting appear to be different for GABAergic and glutamatergic receptors [17].

The aim of this study was to dissect different pathways that Nbea coordinates to target neurotransmitter receptors to the synapse. We used primary neurons from *Nbea* null mutant mice and rescued these neurons using a Nbea mutant that lost affinity for the glutamate receptor interacting protein SAP102. This Nbea mutant is vital for glutamate receptor signalling but not required to support GABA receptor signalling. The Nbea mutant designed to block the previously characterised interaction with PKA II modulates GABAergic and glutamatergic signalling. We conclude that Nbea uses distinct pathways to target glutamate and GABA receptors to the synaptic surface. Understanding these mechanisms may be relevant to the roles of Nbea and SAP102 in humans carrying mutations in these genes that result in idiopathic autism [18] and intellectual disability, respectively [19].

## Methods

### Laboratory Animals, Primary Hippocampal Cultures and Cell Lines

*Nbea* and *SAP102* null mutant mice have been described previously [5,12]. Embryonic day 18 (E18) embryos were obtained by caesarean section of pregnant females through timed heterozygous mating. Hippocampi were dissected from E18 Nbea and SAP102 pups. After removal of meninges, hippocampi were collected in ice-cold Hank’s balanced salt solution (HBSS; Sigma), buffered with 7-mM HEPES (Invitrogen) and further dissociated before plating on glia feeder layer as described previously [15]. For electrophysiology, 25,000 neurons and for immune-cytochemistry, 2000 neurons per 18-mm cover slip were used. For whole-brain lysate, E18 and P84 mice were decapitated; whole brains and different parts of brains were removed and immediately frozen. For immunoblots, brains were boiled in 1X Laemmli Sample buffer. For immunoprecipitation experiments in heterologous cells, human embryonic kidney cells 293T (HEK293T) were used.

### Generation of Nbea Mutant Constructs

Full-length Nbea construct has been described previously [15]. A Nbea construct deficient for binding PKA II was generated by cloning Nbea in two parts: 1-1081aa and 1099-2936aa separately and by joining via restriction sites thereby deleting the 27 amino acid (aa) hypothesised PKA binding site (1081-1099aa) to create PKA mut Nbea (NbeaΔPKA). The SAP102 binding-deficient E2218R mutation was initially created in the C-terminus of Nbea using QuickChange^TM^ Site-Directed Mutagenesis Kit [15]. The C-terminus containing the mutation was re-cloned into full-length Nbea. Phospho-fructo kinase muscle (PFKM) is a putative interactor of Nbea that was identified in mass spectrometry analysis performed as reported previously [15]. Entry clone of human PFKM (Entrez Gene ID: 5213) was a gift from Erich E. Wanker (Max Delbrück Center for Molecular Medicine, Berlin, Germany).

### HEK Cell Transfection, Co-Immunoprecipitation, Immunofluorescence Staining

HEK cell transfection and immunoprecipitation to determine Nbea wild-type (WT) and E2218R binding with SAP102 and PFKM was performed as detailed previously [15]. SAP102 and Nbea WT and KO neurons were allowed to grow till day in vitro (DIV) 14 before fixation. Nbea KO neurons transfected with NbeaΔPKA and E2218R Nbea at DIV 3 were fixed between DIVs 15 and 17. α-SAP102 (mouse monoclonal, NeuroMab clone N19/2, 1:100), α-Nbea (rabbit polyclonal, SySy, 1:1000), α-MAP2 (chicken polyclonal, Abcam ab5392, 1:10000) and α-Actin (mouse monoclonal, Chemicon, 1:200) were used for immunofluorescence staining. Detailed description of staining procedure and Nbea antibody description has been reported previously [15].

### Neuron Transfection and Electrophysiology

Nbea null mutant neurons (25 k/18-mm coverslip) were transfected with WT Nbea, E2218R Nbea or NbeaΔPKA together with enhanced green fluorescent protein (EGFP) as morphology marker at 3 days in vitro (DIV 3) as described previously [20]. Whole-cell voltage clamp recordings were performed at DIVs 14–18 for GABA and glutamate-induced response. Cells were kept in a voltage clamp (membrane potential, *V*_m_ = −70 mV) using Axopatch 200B amplifier (Axon Instruments) with borosilicate glass pipettes (2–4 mOhm) containing 125 mM K^+^-gluconic acid, 10 mM NaCl, 4.6 MgCl_2_, 4 mM K_2_-ATP, 15 mM creatine phosphate (Calbiochem), 1 mM EGTA and 20 U/ml phosphocreatine kinase (pH 7.3, 300 mOsm). All chemicals were from Sigma Aldrich unless otherwise stated. Series resistance was always 70 % compensated and cells with holding current lower than −300 pA were discarded. The external solution contained 140 mM NaCl, 2.4 mM KCl, 4 mM CaCl_2_, 4 mM MgCl_2_, 10 mM HEPES, and 10 mM glucose (pH 7.3, 300 mOsm). Then, 300 nM TTX (Abcam) were added to external medium before recording; 30 μM glutamate or 3 μM GABA was applied by a 20-ms pressure ejection from a glass electrode (~2.8 mOhm). Digidata 1440A and pCLAMP 10 software (Axon instruments) were used to record all signals. All experiments were performed at ambient temperature (20–23 °C).

### RNA Isolation, Real-Time qPCR and Pharmacological Treatment of Cultured Neurons

For RNA isolation, hippocampus of SAP102 WT and KO mice were homogenised in TRIzol reagent and total RNA was isolated using the QIAcube (QIAGEN, Venlo, The Netherlands). The protocol was used according to the manufacturer’s specifications. RNA concentrations and purity were assessed by OD measurements at 260 and 280 nm on a NanoDrop spectrophotometer (Thermo Scientific). For complementary DNA (cDNA) synthesis, 1 μg of RNA and 125 pmol OligoT12 primer dissolved in a total of 10-μl H_2_0 and incubated at 72 °C for 10 min. Reverse transcriptase mix was added, consisting of 5-μl 5X first-strand buffer (Invitrogen), 0.5 μl SuperScript II RNA polymerase (Invitrogen), 10 mM dNTPs, and 25 mM MgCl2 in a total of 15-μl H_2_O. The mixture was incubated at 42 °C for 1 h, followed by 15 min at 70 °C. cDNA quality was assessed on 0.8 % agarose gel. Real-time quantitative PCR (qPCR) was performed using the Light Cycler 480 system (Roche Applied Science, Indianapolis, IN, USA) on SAP102 WT and KO hippocampus. Oligonucleotide primers (Sigma) used for Nbea qPCR were FW: tggctcaaatggagatcaatg and RW: tgtacagctgcttaaagtcacagg. Reaction volumes of 5 μl contained cDNA, 0.1 μM of Universal Probe Library probe 22 (Roche Applied Sciences), 0.4 μM forward primer, 0.4 μM of reverse primer and 2.5 μl 2X LightCycler 480 Probes Master (Roche Applied Science). After initial denaturation for 10 min at 95 °C, amplification was performed using 35 cycles of denaturation (95 °C for 10 s), followed by annealing (58 °C for 15 s) and elongation (72 °C for 15 s). Results were analysed using the LightCycler 480 software (Roche Applied Science) version 1.5. Eukaryotic elongation factor 1-alpha 1 (Eef1a1) is the reference gene to which Nbea messenger RNA (mRNA) levels were normalised to within samples. 0.1 μM of Universal Probe Library probe 31 (Roche Applied Sciences) was used for Eef1a1 gene.

Cycloheximide (CHX) treatments were performed by adding CHX (100 ug/ml Sigma) to cultured mouse cortical neurons at DIV 10 from SAP102 null mice in combination with MG132 (10 μM; Santa Cruz), Leupeptin (25 μM; Sigma). After 24 h, neurons were dissolved directly in 2X SDS sample buffer.

### Image Analysis and Quantification

The GelAnalyzer tool in ImageJ was used to quantify Nbea and SAP102 protein levels in brain lysate. Levels were normalised to valosin-containing protein (VCP) or actin, respectively. Images of neuronal cultures were captured on a Zeiss LSM 510 confocal microscope using a ×40 oil objective (N.A. 1.3) and ×0.7 mechanical zoom and appropriate filter settings. Acquired images were analysed in a semi-automated fashion using MATLAB-based software, SynD [21]. Nbea null neurons expressing WT Nbea, E2218R Nbea or NbeaΔPKA were identified by EGFP. Confocal settings were kept the same for all scans between groups.

### Statistical Tests

Statistical analysis was performed using SPSS (IBM SPSS Statistics 21). The Student’s *t* test was performed taking into account Levene’s test for equality of variances. Kolmogorov-Smirnov and Shapiro-Wilk tests were used to test for normality of data distribution. A Mann-Whitney test for two independent samples was applied when groups were not normally distributed. This study contains nested data, thus when the intra cluster correlation was high (>0.10), which was the case in all electrophysiological data, multilevel modelling was performed to decrease false positives as has been described [22]. The multilevel model was used to find significant difference between three groups (Nbea KO, Nbea KO + WT Nbea, Nbea KO + mutated Nbea) where experimental week was chosen as cluster in which the measurements were nested. When the number of weeks and number of measurements per week allowed, both the intercept and slope where modelled as random coefficients. However, if there was not enough data to fit this complex model, only the intercept was included as a random coefficient when predicting significant differences between groups. Data is represented as mean ± standard error of the mean (SEM). Each condition of each data set has the number of neurons measured over the number of independent experiments/cultures through which the measurements were taken.

## Results

### PKA Binding-Deficient Nbea Rescues Glutamate and GABA Receptor Signalling in Nbea Null Neurons

**Fig. 1:**
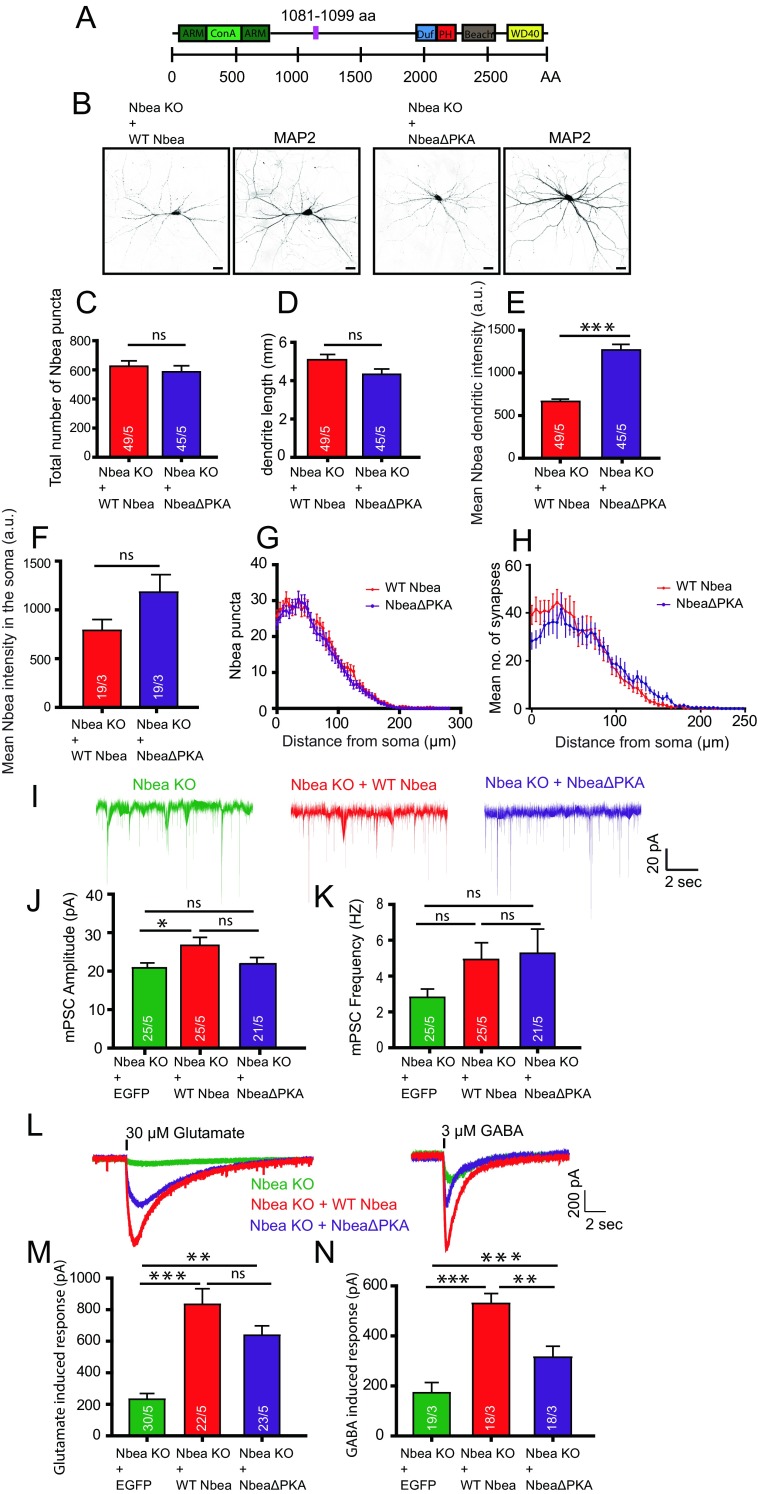
Expression of NbeaΔPKA in Nbea null neurons partially rescues GABA receptor surface expression. **a** Schematic drawing of mouse Nbea showing 22 amino acid deletions to generate NbeaΔPKA. The N-terminus consist concavanalin A (Con A)-like lectin domain surrounded with predicted armadillo (ARM) repeats. The C-terminus has the domain of unknown function 1088 (Duf; in *blue*), the Pleckstrin homology-like domain (PH; in *red*), the Beach domain (*brown*) and the WD40 repeats (*yellow*). Small pink box depicts 1081–1099aa deleted from NbeaΔPKA. **b** Representative image of Nbea KO neurons expressing WT Nbea and NbeaΔPKA depicts similar distribution in cultured neurons. **c** Total number of Nbea puncta in a Nbea KO neuron expressed with WT Nbea and NbeaΔPKA are similar (*n* = 49, 5 independent experiments; *ns* not significant). **d** Dendritic length of Nbea KO neurons rescued with NbeaΔPKA and WT Nbea is not different. **e** Fluorescent intensity of NbeaΔPKA is significantly higher than that of WT Nbea in dendrites (WT Nbea = 666.1 ± 26.52 a.u., *n* = 49, NbeaΔPKA = 1269 ± 65.31, *n* = 45; Mann-Whitney Test, ***p < 0.001). **f** Somatic intensity of NbeaΔPKA is also higher than that of WT Nbea (WT Nbea = 789.5 ± 112 a.u., *n* = 19, NbeaΔPKA = 1182 ± 190.8 a.u., *n* = 19; ns). **g** Sholl analysis shows that NbeaΔPKA distribution is similar compared to WT Nbea. **h** Sholl analysis of synapses in neurons expressing NbeaΔPKA is similar to WT Nbea. **i** Representative trace of spontaneous minis of Nbea KO neurons, Nbea KO rescued with WT Nbea, and Nbea KO rescued with NbeaΔPKA. **j** Amplitude of spontaneous minis of Nbea KO neurons rescued with NbeaΔPKA is insignificant compared to Nbea KO neurons alone (Nbea KO = 20.84 ± 1.302, *n* = 25, Nbea KO + WT Nbea: 26.72 ± 2.074, *n* = 25, Nbea KO + NbeaΔPKA = 21.90 ± 1.635, *n* = 21, 5 independent experiments). **k** Frequency of spontaneous minis of Nbea KO neurons, Nbea KO rescued with NbeaΔPKA, or with WT Nbea are insignificant between groups (Nbea KO = 2.808 ± 0.4671, *n* = 25, Nbea KO + WT Nbea = 4.921 ± 0.9449, *n* = 25, Nbea KO + NbeaΔPKA = 5.268 ± 1.353, *n* = 21, 5 independent experiments). **l** Representative traces of exogenously applied glutamate (30 μM) and GABA (3 μM) on Nbea KO neurons (*green*), Nbea KO rescued with WT Nbea (*red*) and Nbea KO rescued with NbeaΔPKA (*blue*). **m** Expression of WT Nbea and NbeaΔPKA in Nbea KO has significantly higher glutamate-induced responses than Nbea KO neurons. Responses of WT Nbea and NbeaΔPKA neurons are not significantly different (Nbea KO = 231.98 ± 36.99, *n* = 30, Nbea KO + WT Nbea = 833.64 ± 99.42, *n* = 22, Nbea KO + NbeaΔPKA = 638.1 ± 59.82, *n* = 23, 5 independent experiments). **n** GABA induced responses are partially rescued by re-expression of NbeaΔPKA in Nbea null neurons (Nbea KO = 172.75 ± 40.80, *n* = 19, Nbea KO + WT Nbea = 529.56 ± 40.42, *n* = 18, Nbea KO + NbeaΔPKA = 314.96 ± 43.91, *n* = 18, 3 independent experiments). A two-level multilevel statistical analysis was performed including random intercepts. **p < 0.01, ***p < 0.001. All data are mean ± SEM

To test the importance of the Nbea interaction with PKA II in targeting of glutamate and GABA receptors, we generated PKA binding-deficient Nbea (NbeaΔPKA) by deletion of amino acids 1081–1099aa in full-length Nbea as outlined before [23] (Fig. 1a). *Nbea* null mutant (Nbea KO) neurons expressing NbeaΔPKA or wild type Nbea (WT Nbea) showed a similar punctate localization pattern of Nbea in dendrites and the cytoplasm as observed previously for endogenous Nbea [11] (Fig. 1b). Dendritic length, and the number of Nbea puncta in dendrites did not differ between the two groups (Fig. 1c, d). NbeaΔPKA expression was higher in both soma and dendrites compared to WT Nbea (Fig. 1e, f), but the subcellular distribution of Nbea puncta and synapses was similar in both the conditions (Fig. 1g, h). We analysed postsynaptic receptor signalling by application of 30-μM glutamate or 3-μM GABA on Nbea KO neurons expressing WT Nbea or NbeaΔPKA. In line with our previous results [11], expression of WT Nbea increased the amplitude of spontaneous inputs (miniature post synaptic currents, mPSC). This was not observed in neurons expressing NbeaΔPKA (Fig. 1j). mPSC frequency appeared higher in null neurons expressing WT Nbea or NbeaΔPKA but due to the frequency variability this did not reach statistical significance (Fig. 1k). Nbea KO neurons have a strongly reduced response to application of 30-μM glutamate or 3-μM GABA, which can be restored to wild-type levels by re-introduction of WT Nbea [11] (Fig. 1l–n). Compared to Nbea KO neurons rescued with WT Nbea, NbeaΔPKA expressing neurons showed a similar response to glutamate application (Fig. 1l, m). However, the GABA-induced response, although higher than in Nbea KO neurons, was significantly lower in NbeaΔPKA expressing neurons compared to neurons expressing WT Nbea (Fig. 1l, n). Our data shows that PKAII interaction is not required for Nbea function, although rescue appears to be incomplete when expressing the NbeaΔPKA on the null background.

### Nbea Levels are Reduced in SAP102 KO Mice

**Fig. 2:**
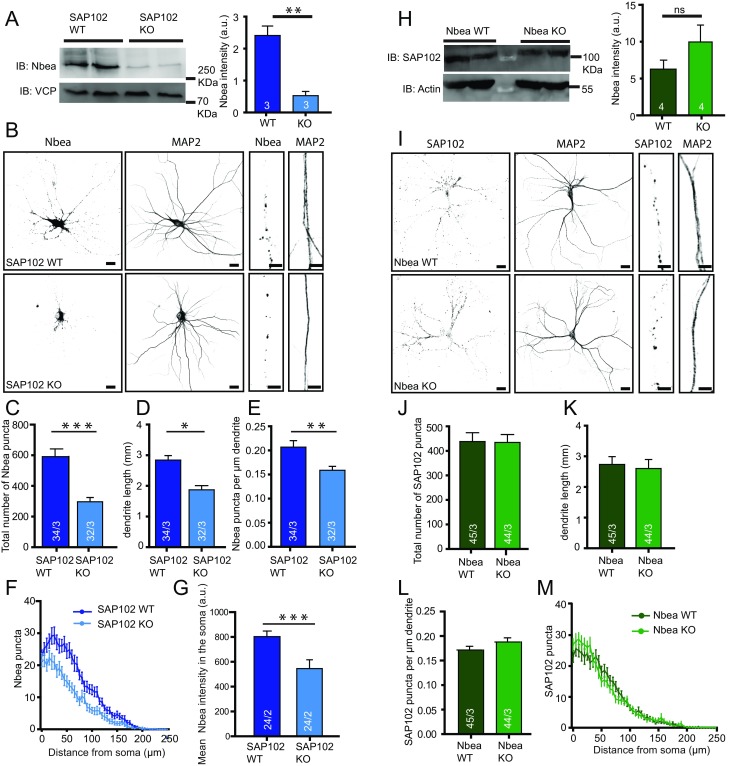
Nbea levels are reduced in E18 SAP102 KO mice. **a** Immunoblot analysis of whole-brain lysates of SAP102 WT and KO mice at E18 for Nbea with VCP as loading control shows fivefold lower Nbea expression in SAP102 KO neurons compared to WT (SAP102 WT = 2.4 ± 0.30 a.u., SAP102 KO = 0.5 ± 0.13 a.u., *n* = 3, Student’s *t* test, *t*(4) = 5.81, *p* = 0.004). **b** Typical images of SAP102 WT and KO cultured neurons show reduction of Nbea puncta in dendrites (*bars* 25 μm, *zoomed image bars* are 5 μm). **c** Nbea is reduced in SAP102 KO-cultured neurons compared to WT. **d** SAP102 KO neurons have shorter dendrites compared to SAP102 WT neurons. **e** Nbea puncta per micrometre dendritic length of SAP102 KO neurons show reduced levels compared to WT. **f** Scholl analysis show altered distribution of Nbea in SAP102 KO compared to WT neurons. **g** Soma analysis show that there is also a significant reduction in levels of Nbea in SAP102 KO neurons (SAP102 WT = 803.3 ± 44.61 a.u., *n* = 24, SAP102 KO = 545.7 ± 70.45 a.u., *n* = 24, Mann-Whitney test, *p* = 0.0003). **h** Western blot analysis of whole brain lysates of Nbea WT and KO mice at E18 for SAP102 with Actin as loading control shows no significant difference (Nbea WT = 9.9 ± 2.2 a.u., Nbea KO = 6.3 ± 1.2 a.u., Student’s *t* test, *t*(6) = 1.42, *p* = 0.21). **i** Typical image of Nbea KO mice show no difference in SAP102 levels (*calibration bars* 25 um, *zoomed image bars* are 5 um). **j** Quantification of SAP102 puncta in Nbea cultured neurons show no difference between KO and WT. **k, l** Dendritic length between Nbea KO and WT neurons is unchanged and SAP102 puncta per micrometre dendritic length is unchanged too. **m** Scholl analysis show unaltered distribution of SAP102 in Nbea WT and KO neurons. *p < 0.05, **p < 0.01, ***p < 0.001. All data are mean ± SEM

SAP102/Dlg3 has been identified as an interactor of Nbea in immunoprecipitation experiments from brain homogenates of embryonic (E18) and adult (P84) mice [15]. To understand the relevance of this interaction, we analysed Nbea levels in *SAP102* null mutant mice (SAP102 KO). Nbea levels in E18 and P84 brain lysates of SAP102 KO mice were significantly reduced (Fig. 2a, Suppl Fig. 1a, b). Quantitative PCR (qPCR) analysis revealed no difference in mRNA levels of Nbea in SAP102 KO neurons compared to SAP102 WT (Suppl Fig. 2a). To further investigate how Nbea is degraded in *SAP102* null mice, we treated SAP102 KO neurons with the protein translation blocker cycloheximide (CHX), proteasome blocker MG132 or lysosome blocker leupeptin. Compared to CHX only, we observed a significant increase in Nbea levels after 24 h of CHX + MG132 but not with the lysosomal blocker leupeptin indicating that Nbea is degraded via ubiquitin-proteasome system (Suppl Fig. 2b, c).

Morphological analysis of cultured SAP102 KO neurons at DIV 14 showed a strong reduction in the number of Nbea puncta in dendrites and intensity of expression in the soma of SAP102 KO neurons (Fig. 2b, c, g). The dendritic length of SAP102 KO neurons was also significantly reduced (Fig. 2d), as well as the number of Nbea puncta per μm dendrite (Fig. 2e). Although Nbea expression is strongly reduced in SAP102 KO neurons, it is still distributed throughout the dendrite (Fig. 2f). In contrast to these SAP102-dependent Nbea phenotypes, in Nbea KO mice, SAP102 levels were unchanged in whole-brain lysates (Fig. 2h), and total numbers of SAP102 puncta, dendritic length and distribution of SAP102 puncta were the same as in WT neurons (Fig. 2j–m). These observations suggest that SAP102 plays an essential role in Nbea expression whereas SAP102 expression is independent of Nbea.

### Glutamate Receptor Signalling Is Unchanged in SAP102 Null Neurons

**Fig. 3:**
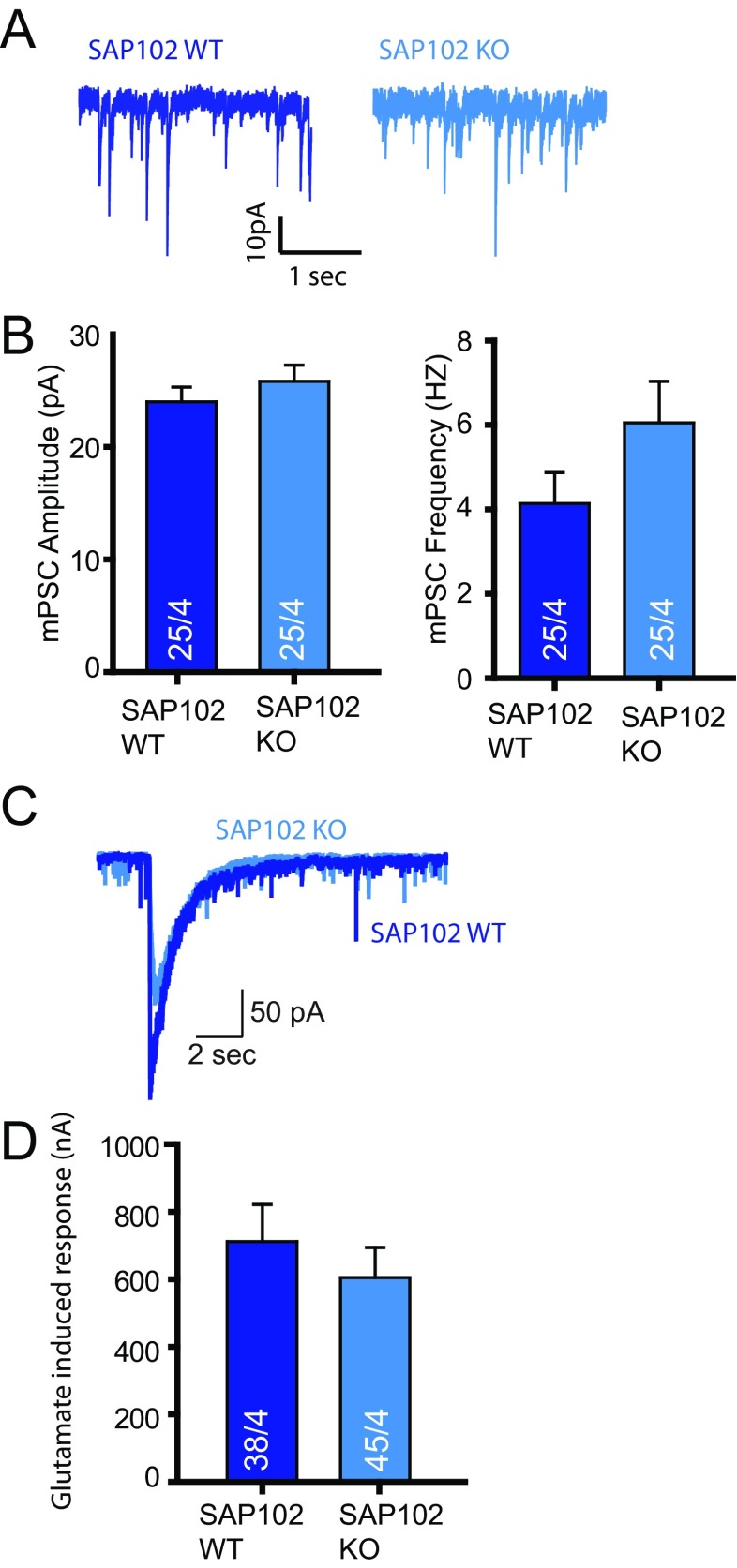
Glutamatergic receptor expression in SAP102 KO neurons is unaltered compared to WT SAP102 neurons. **a** Representative trace of spontaneous minis of SAP102 WT and KO mice. **b** Amplitude and frequency of spontaneous minis of SAP102 WT and KO mice are similar. **c** Representative trace of glutamate (100 uM) application on SAP102 WT and SAP102 KO neurons. **d** Glutamate-induced response on the soma of SAP102 WT and KO mice are not significant from each other (SAP102 WT = 712.4 ± 109.7, *n* = 38, SAP102 KO = 605.0 ± 89.94, *n* = 45, four independent experiments). All data are mean ± SEM

To test whether glutamate receptor signalling is impaired in SAP102 null neurons due to the lower Nbea levels, we recorded spontaneous miniature excitatory postsynaptic currents (mPSCs) in SAP102 WT and KO neurons. Amplitude and frequency were unchanged in SAP102 KO compared to WT (Fig. 3a, b). In addition, exogenous application of 30-μM glutamate showed no significant difference between SAP102 WT and KO neurons (Fig. 3c, d). Hence, despite the ±80 % reduced levels of Nbea in SAP102 null mice, glutamate signalling is unchanged.

### E2218R Nbea Restores Inhibitory Transmission in Nbea Null Neurons

**Fig. 4:**
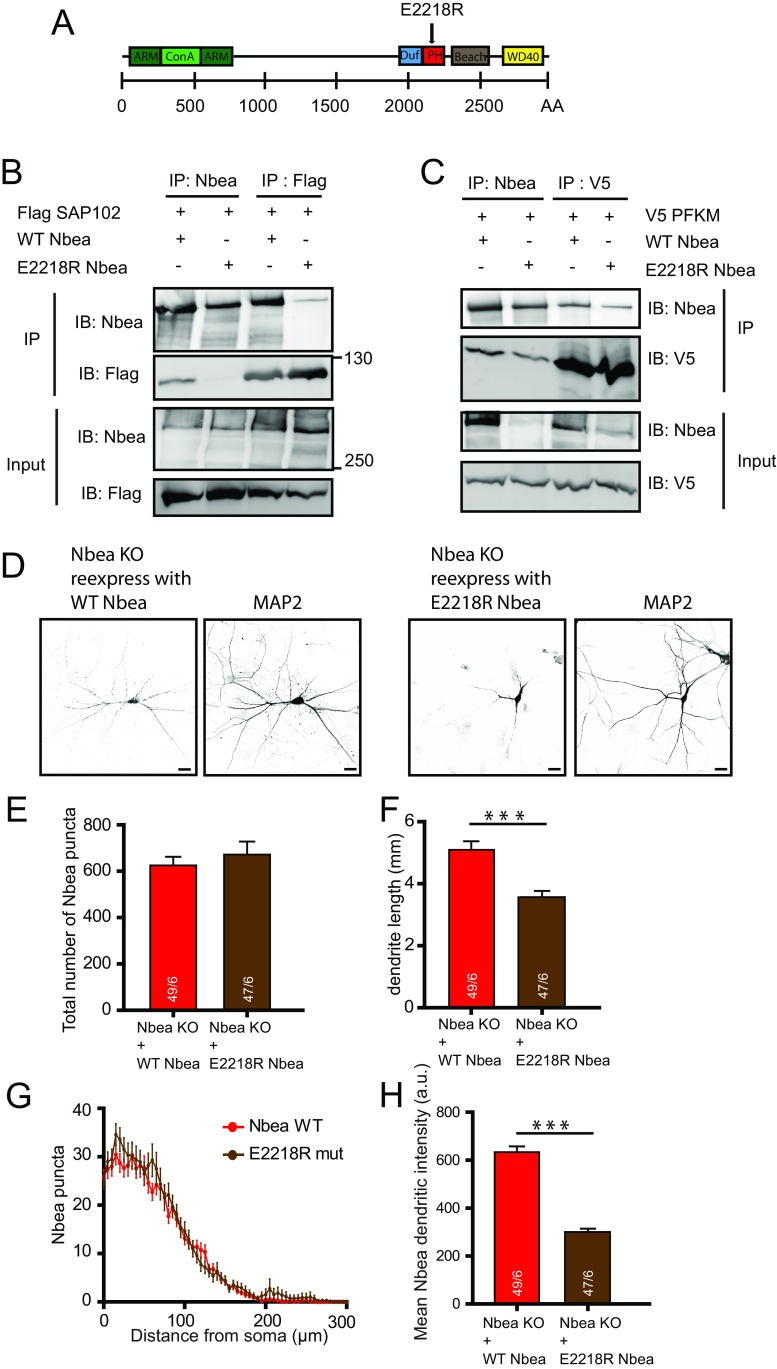
E2218R Nbea does not bind to SAP102 in heterologous cells. **a** Schematic drawing of mouse Nbea and the PH domain in which the E2218R mutation was generated. **b** Immunoblot of a HEK cell IP shows that SAP102 interacts with WT Nbea but does not bind with E2218R Nbea. **c** Immunoblot of a HEK293T cell IP shows that phospho-fructo-kinase (PFKM), another interactor of Nbea, binds with both WT Nbea and E2218R Nbea. **d** Representative image of Nbea KO expressed with WT Nbea and E2218R Nbea depicts similar distribution in cultured neurons. **e** Total number of Nbea puncta in a neuron is not different between WT Nbea and E2218R Nbea. **f** Dendritic length of Nbea KO neurons rescued with E2218R Nbea is shorter than WT Nbea (Nbea KO + WT Nbea: *n* = 49, 5.1 ± 0.3 mm, Nbea KO + E2218R Nbea: *n* = 47, 3.6 ± 0.2 mm, Mann-Whitney test, *U* = 610, ***p < 0.001). **g** Scholl analysis shows that E2218R Nbea distribution in dendrite is same as WT Nbea. **h** E2218R Nbea intensity in dendrites is greatly reduced compared to WT Nbea (Nbea KO + WT Nbea: 633.4 ± 24.13, *n* = 49, Nbea KO + E2218R Nbea: 300.3 ± 13.82 mm, *n* = 47, Mann-Whitney test, *** *p* < 0.001). All data are mean ± SEM

Previously, we have shown that Nbea binds SAP102 via its C-terminus and that a point mutation (E2218R) in the pleckstrin homology (PH) domain of Nbea results in loss of this binding [15]. We engineered this point mutation in full-length Nbea (Fig. 4a) and first performed immunoprecipitation (IP) experiments in HEK cells expressing FLAG-tagged SAP102 and E2218R Nbea to confirm loss of binding (Fig. 4b). Phospho-fructo-kinase (PFKM), a glycolytic enzyme identified in Nbea IPs from mouse brain fractions [15] showed direct binding with WT Nbea when co-expressed in HEK cells (Fig. 4c). This interaction was preserved when IPs were performed with E2218R Nbea. Hence, the E2218R mutation does not result in protein misfolding but specifically loses binding affinity for SAP102. Additionally, we also discovered that WT Nbea and E2218R Nbea bind directly with PSD95 in HEK cells (Sup Fig. 3).

Morphological analysis of Nbea KO neurons expressing WT Nbea or E2218R Nbea showed similar distribution of Nbea in both conditions (Fig. 4d, e, h). Like in the SAP102 null mutant neurons, dendritic length of Nbea KO neurons expressing E2218R Nbea was significantly reduced compared to neurons expressing WT Nbea (Fig. 4f). The intensity of Nbea puncta was reduced in cells expressing E2218R Nbea (Fig. 4g). However, the distribution of WT Nbea and E2218R Nbea puncta was unaltered (Fig. 4h).

**Fig 5:**
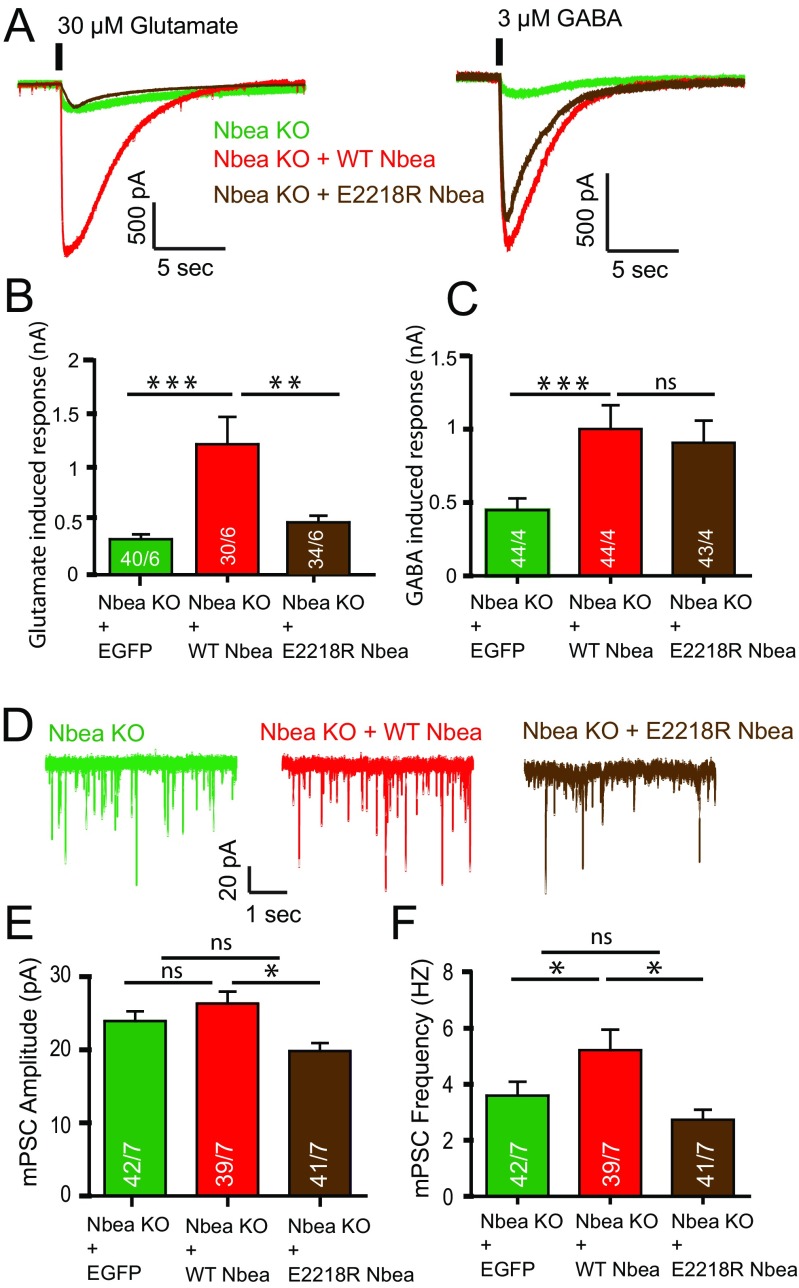
E2218R Nbea does not target Glutamate receptors to the synaptic surface as well as WT Nbea upon expression in Nbea null neurons. **a** Representative trace of exogenously applied glutamate and GABA puff on Nbea KO neurons; Nbea KO rescued with WT Nbea and Nbea KO rescued with E2218R Nbea. **b** E2218R Nbea does not rescue glutamate-induced response as well as WT Nbea in Nbea KO neurons (Nbea KO = 303.56 ± 38.95pA, *n* = 40, Nbea KO + WT Nbea = 917.4 ± 86.15pA, *n* = 30, Nbea KO + E2218R Nbea = 462.3 ± 59.14, *n* = 34, 6 independent experiments). A two-level multilevel statistical analysis was performed including random intercepts. **c** E2218R Nbea rescues GABA induced response as well as WT Nbea in Nbea KO neurons (Nbea KO = 475 ± 84.65pA, *n* = 44, Nbea KO + WT Nbea = 1001.32 ± 162.50pA, *n* = 44, Nbea KO + E2218R Nbea = 908.36 ± 151.55pA, *n* = 43, 4 independent experiments). A two-level multilevel statistical analysis was performed with intercept and slope modelled as random coefficients. **d** Representative trace of spontaneous minis of Nbea KO neurons; Nbea KO rescued with WT Nbea, Nbea KO rescued with E2218R Nbea. **e** Amplitude of spontaneous minis of Nbea KO neurons rescued with E2218R is significantly decreased compared to WT Nbea rescue but non-significant compared to Nbea KO neurons (Nbea KO = 23.31 ± 1.20pA, *n* = 42, Nbea KO + WT Nbea = 25.97 ± 1.48 pA, *n* = 39, Nbea KO + E2218R Nbea = 20.37 ± 0.98pA, *n* = 41, 7 independent experiments). A two-level multilevel statistical analysis was performed including random intercepts. **f** Frequency of spontaneous minis of Nbea KO neurons rescued with E2218R is significantly decreased compared to WT Nbea rescue, whereas non-significant compared to Nbea KO neurons (Nbea KO = 4.04 ± 0.86, *n* = 42, Nbea KO + WT Nbea = 4.92 ± 0.64, *n* = 39, Nbea KO + E2218R Nbea = 3.29 ± 0.47, *n* = 4, 7 independent experiments). A two-level multilevel statistical analysis was performed including random intercepts (***p < 0.001, **p < 0.01, *p < 0.05). All data are mean ± SEM

To investigate the effect of SAP102 interaction on synaptic transmission, we rescued Nbea KO neurons with WT Nbea or E2218R Nbea. Exogenous application of 30-μM glutamate on WT Nbea-expressing neurons showed a complete rescue of synaptic transmission as demonstrated previously [11]. In contrast, glutamate-induced responses in E2218R Nbea expressing neurons were strongly reduced (Fig. 5a, b), whereas 3-μM GABA-induced responses in E2218R Nbea neurons were comparable to null mutants expressing WT Nbea (Fig. 5c). E2218R Nbea neurons showed reduced spontaneous frequency (Fig. 5e) and amplitude (Fig. 5f) compared to WT Nbea rescued neurons. Together, these data suggest that the Nbea-SAP102 interaction is vital for glutamate receptor signalling but not required to support GABA receptor signalling.

## Discussion

In *Nbea* null neurons, AMPA, GABA and NMDA receptors are not targeted and expressed at the synapse surface. Several experiments have demonstrated that this loss of receptor expression is restricted to synapse while extra-synaptic receptor expression is unaffected [11]. Here, we show deletion of hypothesised PKA II binding site in Nbea results in decrease of GABA receptor surface expression (Fig. 1l–n) and the E2218R mutation in Nbea produced a selective loss of glutamate-induced synaptic responses (Fig. 5a–c). SAP102 KO mice have reduced expression of Nbea (Fig. 2a–g) but unaffected glutamate induced synaptic responses (Fig. 3c–d). Conversely, synaptic transmission was impaired in Nbea null mutant neurons even though SAP102 levels were normal (Fig. 2h–m, [11].

### Nbea, as an AKAP, Modulates Receptor Targeting to Synapse

Nbea is an AKAP belonging to a family of BEACH domain proteins. Some of these proteins, such as Lyst or Bchs regulate lysosome-related organelles whereas AKAP550 anchors other proteins [24]. AKAP proteins are of central importance in cellular function due to their ability to interact directly with protein kinases via a common motif at their C-terminus that binds to regulatory subunits of protein kinases and concentrates their intracellular location and regulates their activity [25]. Due to their importance in local signalling events, AKAPs are targets for therapeutic intervention [26]. Nbea interaction with PKA II was found through plasma resonance imaging [16] with a hypothesised 17 amino acid binding site. Deletion of this domain in full length Nbea prevented full rescue of GABA receptor expression on the synapse surface (Fig. 1h, j). This suggests that anchoring and/or concentrating PKA II is important for the insertion of GABA receptors (although a trend towards incomplete rescue of glutamate-induced responses was also observed, Fig. 1m). Protein kinase A is known to regulate inhibitory receptors such as glycine receptor-induced Cl^−^ currents by increasing the probability of channel openings [27]. Reduction of mPSC amplitude and mPSC frequency in Nbea KO can be rescued with re-introduction of WT Nbea [11]. We do not see any difference in the mPSC amplitude and frequency of neurons rescued with NbeaΔPKA compared to the other two groups. Our data show that Nbea essentially does not require PKA binding to perform its function but this interaction modulates inhibitory and excitatory receptor surface expression.

### E2218R Nbea Mutant Suggests That SAP102 Binding to Nbea Is Essential for Excitatory but not Inhibitory Transmission

Expression of the E2218R Nbea mutant in Nbea KO neurons did not restore glutamate receptor expression at the synapse surface (Fig. 5a, b). Additionally, significantly reduced mPSC amplitude and frequency in neurons rescued with E2218R Nbea compared to WT Nbea rescue suggests reduced expression of synaptic receptors (Fig. 5d–f). As AKAP-MAGUK-NMDA receptor complexes are known to exist [28], we predicted that Nbea indirectly interacts with NMDA receptors via SAP102 to target receptors to the synapse surface. This mechanism might be similar to the SAP97/Dlg1 interaction with AKAP97, which is disrupted by calmodulin-dependent protein kinase II resulting in a decrease of GluR1-mediated AMPA receptor currents [29]. Nbea KO neurons expressed with E2218R Nbea have shorter dendritic length than neurons with WT Nbea (Fig. 4f), which is a phenocopy of shorter dendrites present in SAP102 KO neurons (Fig. 2d). We did not observe changes in glutamate-induced responses in SAP102 KO neurons (Fig. 3c, d). PSD95 interaction with WT Nbea and E2218R Nbea (Suppl Fig. 3) suggests that other MAGUK/Dlgs maybe taking over the function of SAP102 in SAP102 null mice, potentially via the promiscuous PDZ-binding ligand found in all NR2 subunits of the NMDA receptors [30,31]. Overlapping functions of PSD95, PSD93 and SAP102 in transport of glutamate receptor trafficking has been documented, in which only knocking out two or three MAGUKs results in severe loss of receptor signalling [32]. Additionally, both PSD95 and SAP102 knockout mice show enhanced hippocampal long-term potentiation consistent with a similar signalling function [33]. Developmental switches and redundant functions among different MAGUKs [4,34] might explain the fact that the synaptic machinery is intact in SAP102 KO mice. In line with this idea, acute SAP102 knock down in neurons produced unique phenotypes, not observed upon chronic deletion of SAP102 expression in null mutant mice [35]. These phenotypes, for instance a reduced excitatory synaptogenesis [35] point in the same direction as morphological and functional changes observed when the interaction between SAP102 and Nbea is prevented (using the E2218R mutant).

### Cellular Nbea Stability Depends on SAP102

Analysis of *Nbea* null brain homogenate and cultured neurons showed no difference in cellular levels in many pre- and post synaptic proteins [11], while synapsin, synaptophysin and Mint1 were found to differentially expressed in the brain stem of *Nbea* null mice [14]. Our observation that SAP102 protein levels are unchanged in *Nbea* null mice demonstrates that loss of Nbea does not have a general effect on stability or transportation of other proteins in cultured neurons. However, Nbea protein levels are greatly reduced in *SAP102* null neurons and brain homogenate (Fig. 2a–g). The Nbea mutant that abolished SAP102 binding showed a lower staining intensity when expressed in null mutant neurons than the wild type Nbea protein (Fig. 4h). Nbea mRNA levels are unchanged in *SAP102* null neurons (Suppl Fig. 2a). This suggests that SAP102 regulates stability of Nbea protein and we show that in the absence of SAP102, Nbea protein is degraded via the ubiquitin-proteasome pathway (Suppl Fig. 2b, c). The 80 % reduction in Nbea protein levels at E18 still results in normal synaptic transmission in SAP102 KO mice (Fig. 3c, d).

### Functional Changes in *Nbea* Null Synapses Might Have Structural Consequences

We observe changes in synaptic surface expression of receptors in *Nbea* null neurons. Functional changes can lead to structural changes in neuronal network. Cultured *Nbea* null neurons from the brain stem have an impaired development and function of spinous synapses with an accumulation of F-actin and synaptopodin [13]. Shorter spine density is possibly due to decrease in surface expression of post-synaptic receptors on the synapse surface that we observe due to Nbea loss.

### Nbea Targets Excitatory and Inhibitory Post-Synaptic Receptors via Distinct Pathways

Nbea immunoreactivity was found to be apposed to certain inhibitory synapses [36] juxtaposed to the Golgi complex and accumulated in endosome-like organelles in dendrites [11]. Hence, Nbea resides probably at locations in the cell where receptors and their interacting proteins assemble for targeting to the synaptic membrane. The interaction between Nbea, PKAII and SAP102 might also take place at such locations, upstream of insertion of receptors at the synapse surface. To our knowledge, Nbea is the first protein that regulates synaptic receptor expression to both excitatory and inhibitory synapses. The current data now demonstrate that Nbea orchestrates these trafficking routes using distinct pathways, one resistant to the loss of SAP102-NBea interaction (GABA-receptor targeting) and one that is not supported by the Nbea mutant that abolishes this interaction (glutamate receptor targeting).

## Electronic supplementary material

Below is the link to the electronic supplementary material.

Suppl Fig 1 Nbea levels are reduced in P84 SAP102 KO mice. **a** Immunoblot analysis of whole brain lysates of SAP102 WT and KO mice at P84 for Nbea with VCP as loading control shows twofold lower Nbea expression in SAP102 KO neurons compared to WT (SAP102 WT = 6.2 ± 0.49 a.u., SAP102 KO = 3.3 ± 0.33 a.u., *n* = 2, Student’s *t* test, *t*(2) = 4.82, *p* = 0.041). **b** Immunoblot analysis of cortex, hippocampus and cerebellum of SAP102 WT and KO mice at P84 for Nbea with VCP as loading control shows twofold lower Nbea expression in SAP102 KO neurons compared to WT (SAP102 WT cortex = 6.71 ± 0.35 a.u., SAP102 KO cortex = 3.97 ± 0.49 a.u., *n* = 2, Student’s *t* test, *t*(2) = 4.51, *p* = 0.045. SAP102 WT hippocampus = 6.38 ± 0.19 a.u., SAP102 KO hippocampus = 3.27 ± 0.25 a.u., *n* = 2, Student’s *t* test, *t*(2) = 9.87, *p* = 0.01. SAP102 WT cerebellum = 6.33 ± 0.43 a.u., SAP102 KO cerebellum = 3.29 ± 0.30 a.u., *n* = 2, Student’s *t* test, *t*(2) = 5.84, *p* = 0.045). (*p < 0.05). All data are mean ±SEM (GIF 67 kb)

High-resolution image (EPS 835 kb)

Suppl Fig 2 Nbea mRNA transcription or protein stability is not affected due to loss of SAP102. **a** Nbea mRNA levels are not significantly different between E18 SAP102 WT and KO mice. **b** Cortical neurons (at DIV 10) from *SAP102* null mice were incubated for 24 h with cycloheximide (CHX), or in combination with MG132 or leupeptin (Leu) (*n* = 2). Munc-13 is known to be degraded by ubiquitin-proteasome pathway and is used as a positive control. VCP is used as a loading control as it has a longer half-life and is not greatly affected by 24-h drug treatment. **c** MG132 addition but not leupeptin rescues Nbea expression levels compared to CHX only demonstrating that Nbea is degraded by ubiquitin-protsome pathway (**p < 0.01), (*p < 0.05) All data are normalised to *t* = 0 condition. (GIF 82 kb)

High-resolution image (EPS 865 kb)

Suppl Fig 3 PSD95 interacts with WT Nbea and E2218R Nbea in heterologous cells. **a** Immunoblot of a HEK cell IP shows that PSD95 interacts with WT Nbea and E2218R Nbea in HEK cells (GIF 24 kb)

High-resolution image (EPS 499 kb)
