# Supplementary figures and images for: Neurobeachin Regulates Glutamate- and GABA-Receptor Targeting to Synapses via Distinct Pathways
Source: Mol Neurobiol. 2015 May 2;53:2112–23. doi: 10.1007/s12035-015-9164-8 (PMC4823379; doi:10.1007/s12035-015-9164-8)

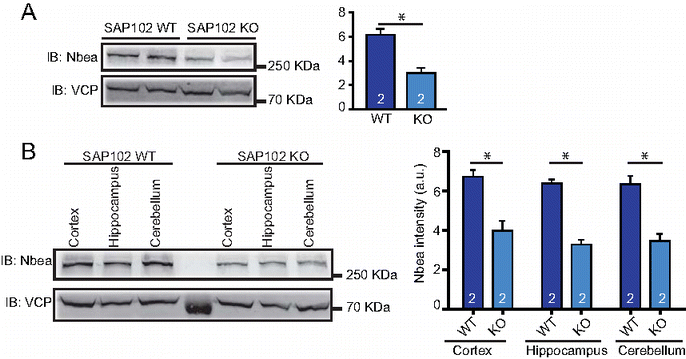

Supplement: Supplementary file 1 — Nbea levels are reduced in P84 SAP102 KO mice. a Immunoblot analysis of whole brain lysates of SAP102 WT and KO mice at P84 for Nbea with VCP as loading control shows twofold lower Nbea expression in SAP102 KO neurons compared to WT (SAP102 WT = 6.2 ± 0.49 a.u., SAP102 KO = 3.3 ± 0.33 a.u., n = 2, Student’s t test, t(2) = 4.82, p = 0.041). b Immunoblot analysis of cortex, hippocampus and cerebellum of SAP102 WT and KO mice at P84 for Nbea with VCP as loading control shows twofold lower Nbea expression in SAP102 KO neurons compared to WT (SAP102 WT cortex = 6.71 ± 0.35 a.u., SAP102 KO cortex = 3.97 ± 0.49 a.u., n = 2, Student’s t test, t(2) = 4.51, p = 0.045. SAP102 WT hippocampus = 6.38 ± 0.19 a.u., SAP102 KO hippocampus = 3.27 ± 0.25 a.u., n = 2, Student’s t test, t(2) = 9.87, p = 0.01. SAP102 WT cerebellum = 6.33 ± 0.43 a.u., SAP102 KO cerebellum = 3.29 ± 0.30 a.u., n = 2, Student’s t test, t(2) = 5.84, p = 0.045). (*p < 0.05). All data are mean ±SEM (GIF 67 kb) [file 12035_2015_9164_Fig6_ESM.gif]

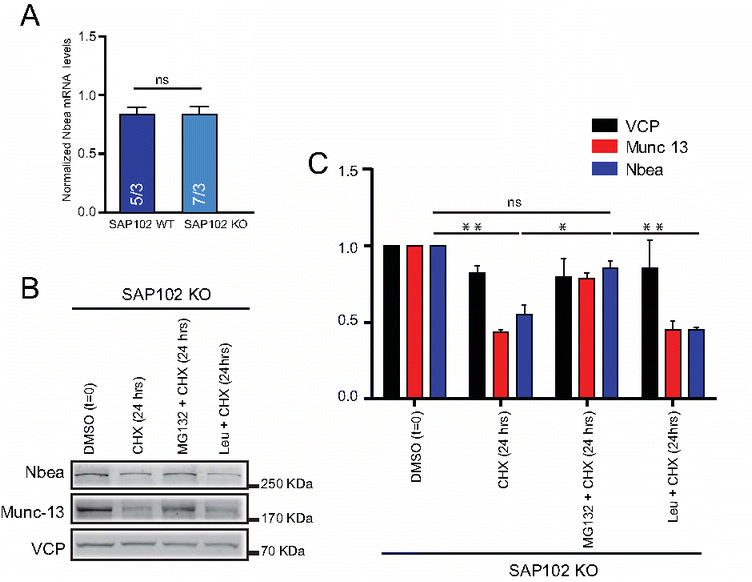

Supplement: Supplementary file 3 — Nbea mRNA transcription or protein stability is not affected due to loss of SAP102. a Nbea mRNA levels are not significantly different between E18 SAP102 WT and KO mice. b Cortical neurons (at DIV 10) from SAP102 null mice were incubated for 24 h with cycloheximide (CHX), or in combination with MG132 or leupeptin (Leu) (n = 2). Munc-13 is known to be degraded by ubiquitin-proteasome pathway and is used as a positive control. VCP is used as a loading control as it has a longer half-life and is not greatly affected by 24-h drug treatment. c MG132 addition but not leupeptin rescues Nbea expression levels compared to CHX only demonstrating that Nbea is degraded by ubiquitin-protsome pathway (**p < 0.01), (*p < 0.05) All data are normalised to t = 0 condition. (GIF 82 kb) [file 12035_2015_9164_Fig7_ESM.gif]

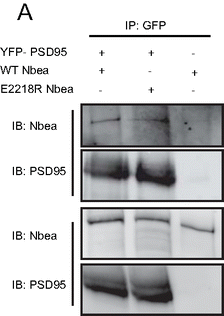

Supplement: Supplementary file 5 — PSD95 interacts with WT Nbea and E2218R Nbea in heterologous cells. a Immunoblot of a HEK cell IP shows that PSD95 interacts with WT Nbea and E2218R Nbea in HEK cells (GIF 24 kb) [file 12035_2015_9164_Fig8_ESM.gif]
